# Terahertz spectroscopy study of oridonin and ponicidin in the anticancer Chinese herbal medicine Rabdosia rubescens

**DOI:** 10.3389/fpls.2024.1460123

**Published:** 2024-10-29

**Authors:** Yang Gao, Zhuang Peng, Huiyu Yang, Xinrui Zhang, Yuhan Zhao, Zeyu Hou, Bo Su, Kai Li, Cunlin Zhang

**Affiliations:** ^1^ Key Laboratory of Terahertz Optoelectronics, Ministry of Education, Beijing, China; ^2^ Beijing Key Laboratory for Terahertz Spectroscopy and Imaging, Capital Normal University, Beijing, China; ^3^ Beijing Advanced Innovation Center for Imaging Theory and Technology, Capital Normal University, Beijing, China; ^4^ Department of Physics, Capital Normal University, Beijing, China

**Keywords:** terahertz, oridonin, ponicidin, Raman spectroscopy, PXRD

## Abstract

Rabdosia rubescens, a Chinese herbal medicine with anticancer properties, contains two active ingredients: oridonin and ponicidin. Both compounds exhibit antitumor effects by inducing tumor cell apoptosis and autophagy and inhibiting tumor cell proliferation. To further explore the differences in molecular structure and pharmacological properties between the two substances, this study employs Terahertz Time-Domain Spectroscopy (THz-TDS) to investigate the spectral characteristics of oridonin and ponicidin in the frequency range of 0.1 to 2.3 THz. The crystal structures of the two substances are simulated using Materials Studio software and Density Functional Theory (DFT), yielding their spectra and molecular vibration modes, which elucidate the mechanism underlying the peak generation. The consistency between experimental and simulation results confirms the reliability of the experimental findings. Thus, THz spectroscopy can effectively distinguish between these two substances. Finally, a THz detection is performed on Rabdosia rubescens capsules purchased from the market, revealing the presence of absorption peaks for both substances in their absorption spectra. This provides a new approach for detecting active ingredients in Chinese herbal medicines.

## 1 Introduction

There are various methods to detect the biochemical properties of different biomolecules, such as Raman spectroscopy, Infrared Spectroscopy, and X-ray diffraction (Pfeiffer, 2012; Finneran et al., 2016). Although these methods have many advantages, they all suffer from issues like operational complexity and limited sensitivity. In recent years, Terahertz Time-Domain Spectroscopy (THz-TDS) technology has developed rapidly and has been widely used in various fields of medicine and biology. THz-TDS can provide sufficient optical properties of molecular-level interactions by acquiring information such as the amplitude and phase of biomolecules (Guo et al., 2020). In addition, THz is an electromagnetic wave with a frequency range of 0.1 to 10 THz and a wavelength range of 30 to 3000 μm. Its waveband can cover the characteristic spectra of semiconductors, plasmas, organisms, biological macromolecules, and other substances. Using this frequency band can deepen and expand human understanding of some fundamental scientific issues in physics, chemistry, astronomy, informatics, and life sciences, providing a powerful means for further research on biochemical reactions.

Rabdosia rubescens, also known as Blood-breaking Pill, Ice Sweet, etc., belongs to the genus Isodon of the Labiatae family. It is widely distributed in the Yellow River and Yangtze River basins, with the main producing area being Jiyuan, Henan Province, China. Due to its significant resource advantages and good pharmacological activity, Rabdosia rubescens has become a commonly used Chinese herbal medicine. In 1991, the Ministry of Health of China approved the dried leaves and aboveground parts of Rabdosia rubescens as Chinese medicinal materials, which were included in the standards for Chinese medicinal materials issued by the Ministry of Health. Because of its anti-inflammatory and anti-tumor properties, scientific researchers have conducted studies on its chemical composition, pharmacological effects, and clinical applications (Hao and Guo, 2023), discovering a large number of diterpenoids. Among them, oridonin and ponicidin are tetracyclic diterpenoid anti-cancer active ingredients isolated from Rabdosia rubescens. Oridonin, with a chemical formula of C_20_H_28_O_6_ and a molecular weight of 364.433, appears as a pale yellow needle-like crystal that is slightly soluble in water but can be dissolved in organic solvents such as methanol, ethyl acetate, and ethanol. Ponicidin, with a chemical formula of C_20_H_26_O_6_ and a molecular weight of 362.420, exists as a white crystalline powder soluble in organic solvents like methanol, ethanol, and DMSO. In their chemical structures, the conjugation of cyclopentyl ketone and exocyclic methylene serves as the center of physiological activity (Chen et al., 1987). Molecular inactivation occurs when the ring splits or the methylene group becomes saturated. Research findings indicate that oridonin exerts a significant inhibitory effect on tumors through various pathways, including inducing tumor cell apoptosis and autophagy, inhibiting tumor cell proliferation, invasion, and migration, altering tumor cell drug resistance, and reducing telomerase activity (Xu et al., 2020). Oridonin can initiate cancer cell apoptosis mechanisms via different anti-tumor mechanisms for various types of cancer cells, such as human laryngeal cancer Hep-2 cells, acute promyelocytic leukemia cells, and liver cells, thereby effectively controlling cancer cell growth and reproduction. It is widely used in the treatment of liver cancer, esophageal cancer, and pancreatic cancer, and has achieved certain clinical efficacy (Hsieh et al., 2005). The chemical structure and pharmacological activity of ponicidin bear similarities to oridonin, both possessing anticancer effects. Relevant experiments have demonstrated that oridonin exhibits a smaller toxic effect on normal blood cells surrounding tumor cells when inducing tumor cell apoptosis, whereas ponicidin shows a stronger anticancer activity in various cancers, including colorectal cancer, by inhibiting protein kinase pathways to hinder cancer cell infiltration and metastasis. Additionally, ponicidin has a certain stimulating effect on cellular immunity, which distinguishes it from oridonin. Given the anticancer effects and wide clinical applications of oridonin and ponicidin, understanding their basic properties, including spectral characteristics, is crucial. Ke et al. (2010) employed UV spectrophotometry to determine the total diterpene content in Rabdosia rubescens extracts, with easy experimental control and stable, reliable results. Zhang et al. (2011) utilized the Waters UPLC chromatographic system to complete the chromatographic analysis of five components: oridonin, ponicidin, rosmarinic acid, oleanolic acid, and ursolic acid, achieving good separation between the chromatographic peaks of each component. Currently, there are limited methods for determining the content of Rabdosia rubescens, with UV spectrophotometry and high-performance liquid chromatography (HPLC) being reported in the literature (Zhang and Tu, 2020). While HPLC offers high separation efficiency, fast analysis speed, and high detection sensitivity, it also has drawbacks such as high equipment costs, significant consumption of the mobile phase, and demanding sample pretreatment requirements. Therefore, we consider using THz spectroscopy to analyze the two substances, which provides high resolution, safety, and eliminates the need for complex sample pretreatment. This paper employs THz-TDS system to investigate the spectral characteristics of oridonin and ponicidin in the frequency range of 0.1 to 2.3 THz. The use of THz spectroscopy allows for effective differentiation between the two substances.

This paper focuses on the study of the main active substances in Rabdosia rubescens, namely oridonin and ponicidin. Initially, the THz absorption spectra of these two compounds in solid state were measured within the frequency range of 0.1 to 2.3 THz. Following this, molecular dynamics simulations were conducted, and the absorption peak information of the samples was calculated through data processing. Additionally, DFT analysis was performed to investigate the absorption characteristics and origins of their THz absorption spectra from the perspective of molecular vibrations. The simulation studies primarily utilized the CASTEP software package and PBE density functional, yielding simulation results that aligned with the experimental findings, thus confirming the reliability of the experiment. Simultaneously, DFT simulations provided deeper insights into the intermolecular interactions, which are the primary factors contributing to the appearance of absorption peaks in the THz region. Both compounds exhibited similar absorption peaks at 1.76 and 2.14 THz in their THz absorption spectra, attributable to their shared structure featuring a conjugated cyclopentanone and exocyclic methylene group. However, due to slight differences in their molecular structures, there were distinctions in their THz absorption spectra. Oridonin exhibited an absorption peak at 1.55 THz, whereas ponicidin displayed three distinct peaks at 1.85, 1.96, and 2.22 THz. This allowed for the effective differentiation of these two compounds using THz-TDS. Through DFT analysis of the self-constructed unit cell structures of both substances, the reasons for the emergence of different absorption peaks in the THz region were elucidated. Furthermore, a comparison between the simulated and experimental results of X-ray diffraction in the solid state for both compounds revealed a high degree of consistency. This further validated the reliability of the THz simulation results obtained through DFT analysis of the self-constructed unit cells. Additionally, the THz absorption spectra of the two substances in ethanol solution were investigated, revealing a correlation with their solid-state THz absorption spectra. Notably, some absorption peaks underwent changes, indicating that the intramolecular and intermolecular interactions of oridonin and ponicidin were weakened by water molecules. Finally, common Rabdosia rubescens Capsules available in the market were subjected to THz detection. The detection results revealed the respective absorption peaks of oridonin and ponicidin, demonstrating that THz detection can identify these compounds within the capsules. This paves the way for quantitative analysis of oridonin and ponicidin in pharmaceutical products, and lays a foundation for more objective and effective quality control and evaluation of Rabdosia rubescens medications using THz techniques.

## 2 Materials and methods

### 2.1 Terahertz time-domain spectroscopy system

**Figure 1:**
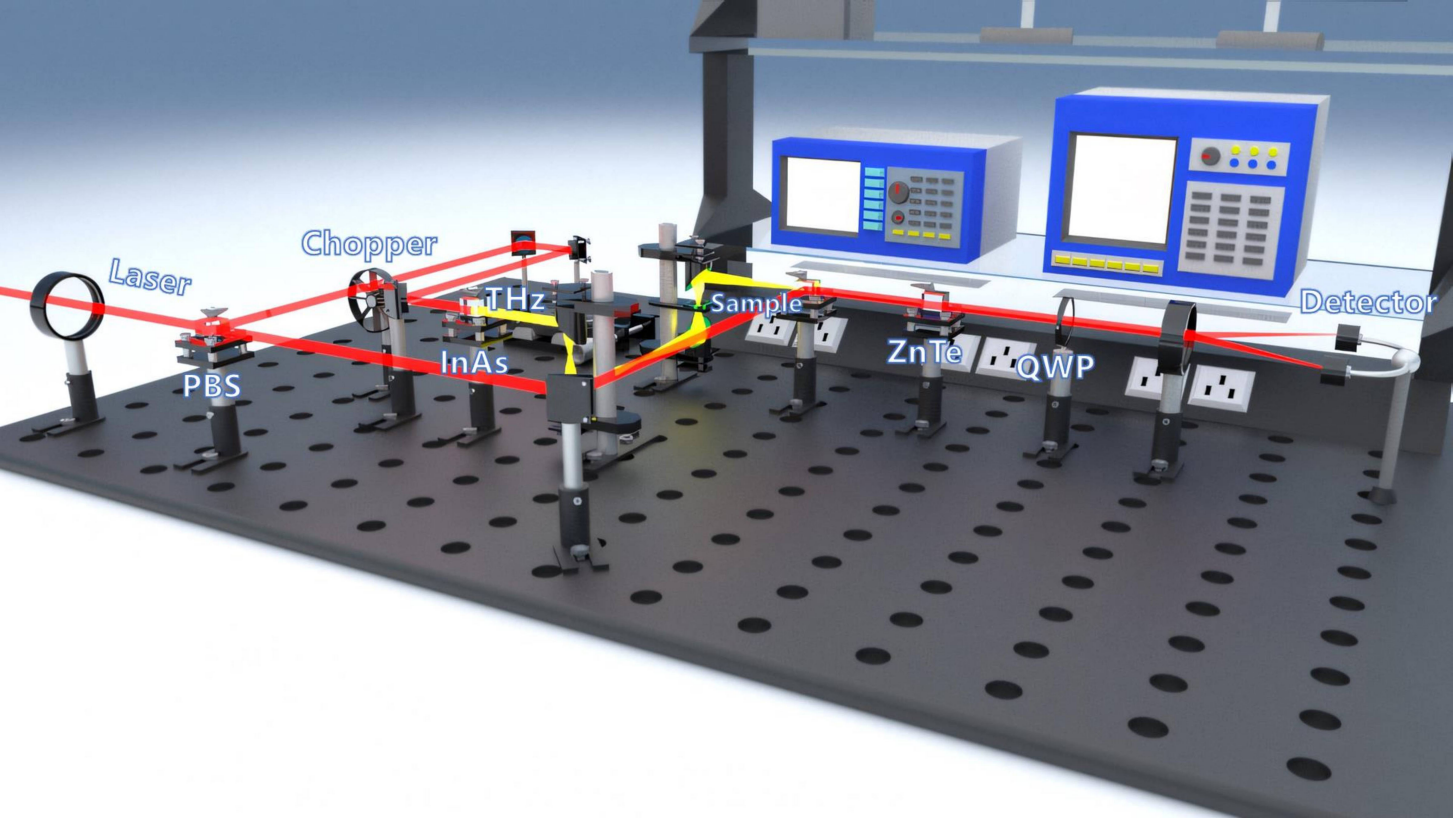
Experimental optical path diagram.

In this study, a self-built transmissive THz-TDS system was employed, consisting of a Maitai femtosecond laser, a chopper, and devices for generating and detecting THz waves, as illustrated in **Figure 1**. The Maitai femtosecond laser, a titanium sapphire laser, operates at a central wavelength of 800 nm, a repetition rate of 82 MHz, and an output power of 3.4 W. The laser pulses, modulated by a C-995 chopper and synchronized with a lock-in amplifier, are focused onto an InAs crystal through a lens and an electric translation stage, generating THz pulses via radiation. These pulses, after collimation and focusing by a parabolic mirror, are re-collimated by another parabolic mirror and directed into a ZnTe crystal. Simultaneously, a femtosecond probe beam reaches the ZnTe crystal, where it undergoes electro-optic modulation to alter its polarization state. The modified beam passes through a Wollaston prism and is then detected by a differential detector. The resulting weak current is fed into a lock-in amplifier for amplification, shaping, and phase adjustment. This process effectively enhances the signal, suppresses incoherent noise, achieving a signal-to-noise ratio exceeding 800, and covers a spectral range of 0.2-2.3 THz. The system operates with a scanning step size of 1 μm, a scanning length of 3 cm, a frequency domain resolution of 37 GHz, and a time domain resolution of 66 fs.

### 2.2 Samples

**Figure 2:**
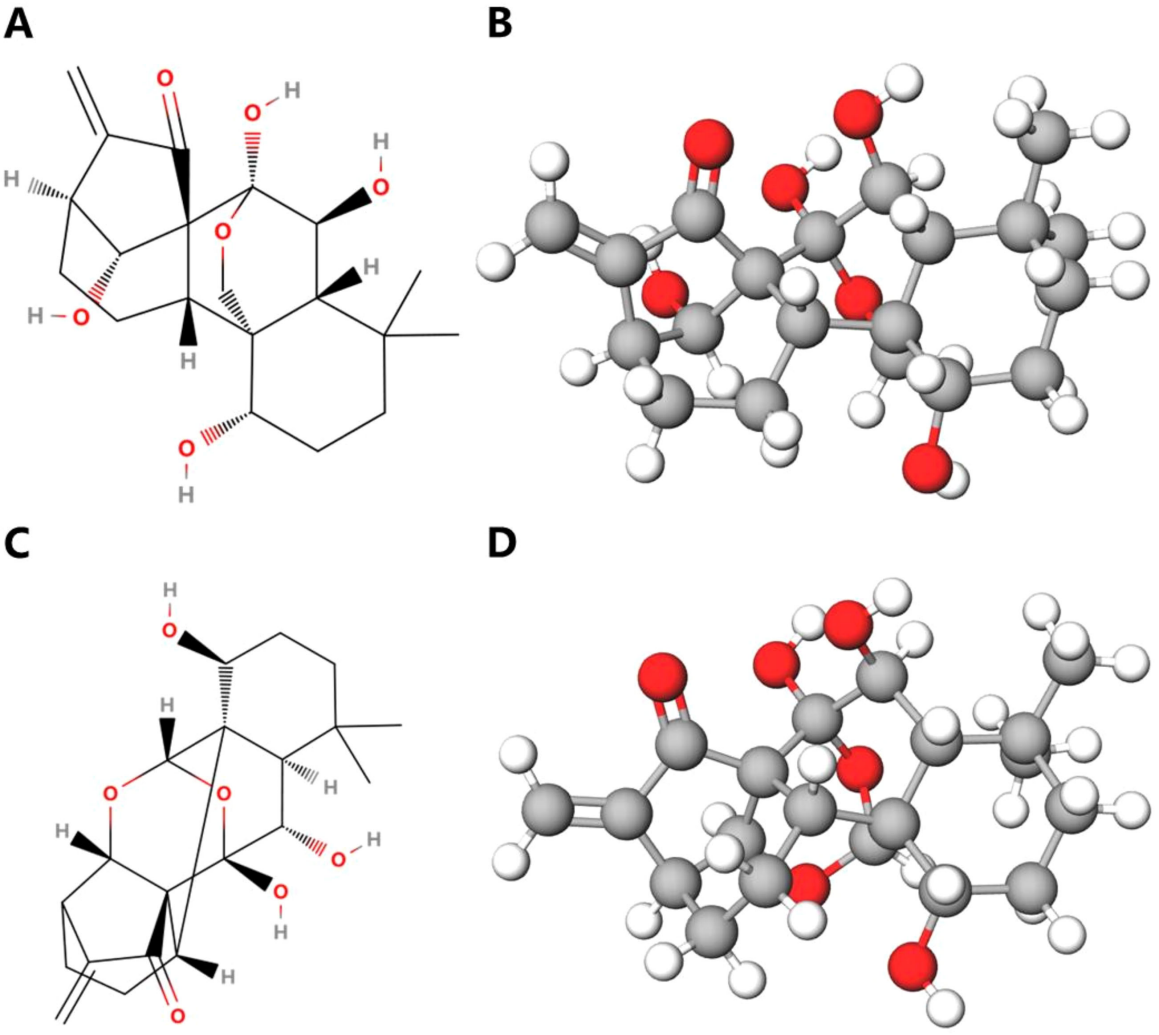
The chemical structures of oridonin and ponicidin. **(A)** Planar graph of oridonin molecule. **(B)** Three-dimensional diagram of oridonin molecule. **(C)** Planar graph of ponicidin molecule. **(D)** Three-dimensional diagram of ponicidin molecule.

Oridonin (98% purity) was purchased from Xi’an Haoxuan Biotechnology Co., Ltd. in China, appearing as a white crystalline powder; while ponicidin (98% purity) was sourced from Nanjing Yuanzhi Biotechnology Co., Ltd. in China, presenting as a light-yellow needle-like crystal. The Rabdosia rubescens Capsule used was Wangwushan Rabdosia rubescens Capsule, produced by Jishi Pharmaceutical in Jiyuan City, Henan Province, China. The three samples were mixed and pulverized with polyethylene powder (PET) at a mass ratio of 125:25 mg, and then pressed under a pressure of 6 Mpa for 5 minutes to obtain round tablets with a thickness of 1.1 mm. Afterwards, oridonin and ponicidin were mixed with ethanol solution at their maximum saturation, resulting in oridonin-ethanol solution and ponicidin-ethanol solution, respectively. The molecular structures of the two substances are shown in **Figure 2**.

### 2.3 Microfluidic chip device

**Figure 3:**
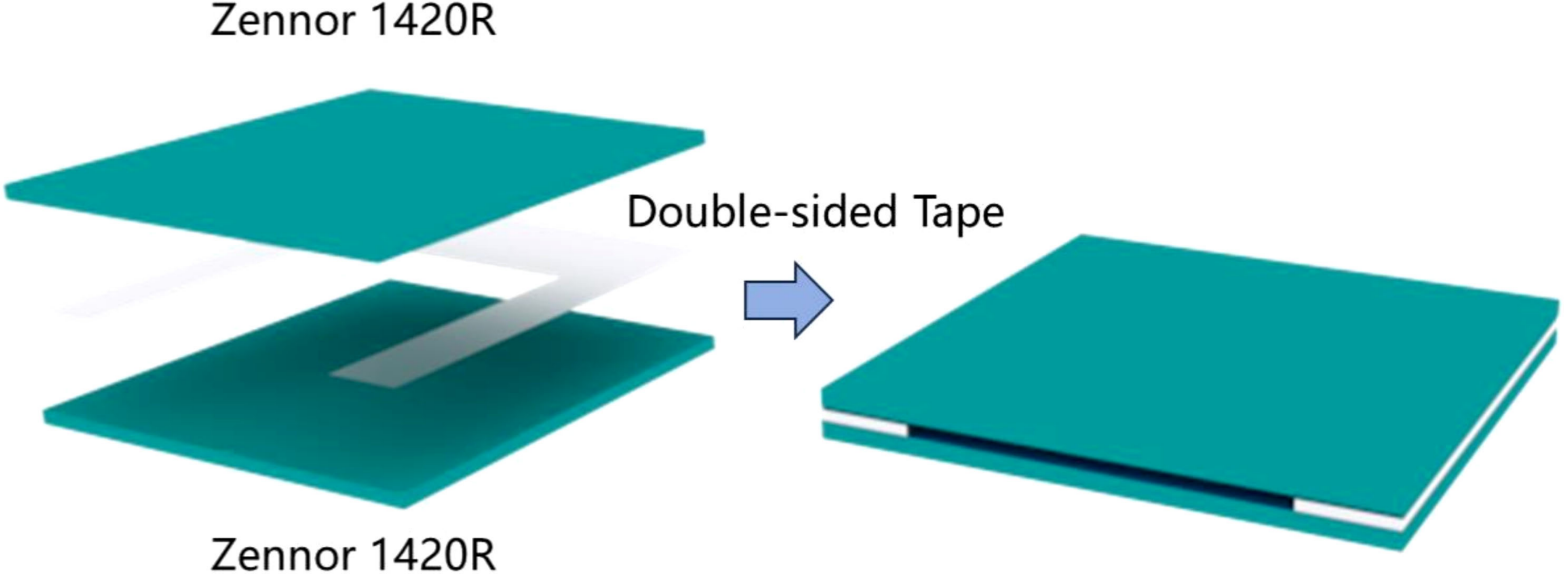
Microfluidic chip.

Our research group often uses cyclic olefin copolymer (COC) to fabricate microfluidic chips for studying the THz transmission characteristics of liquid samples. This is because COC exhibits high transmission properties for THz waves, with a THz transmittance rate of up to 95%. Additionally, its transparency to visible light makes it an ideal material for the preparation of microfluidic chips (Zhong et al., 2019). In this experiment, two pieces of COC, each with dimensions of 3 cm × 3 cm × 2 mm, were used as the substrate and cover. A square area with a length and width of 2 cm was etched onto a 50 μm thick 3M double-sided adhesive tape. The tape was then bonded to the substrate and cover to create the microfluidic chip. The fabrication process is illustrated in **Figure 3**. This design allows fluid to flow in and out of the slit at the top of the chip, making it more convenient and efficient during sample injection and removal. The chip can be reused by injecting deionized water through the side for cleaning.

### 2.4 Calculation method

In this experiment, to eliminate the influence of Fabry-Perot oscillations, we adopted a slab medium model based on Fresnel’s formulas for data processing. The focus is on using a physical model capable of extracting the THz optical parameters of materials to obtain the absorption coefficient of the sample (Duvillaret et al., 1999; Dorney et al., 2001). This model performs Fourier transforms on the THz time-domain waveforms of both the reference and transmitted samples to acquire amplitude and phase information. It can calculate the refractive index of a sample with a known thickness *d*.


(1) $$n(\omega )=\frac{c}{\omega d}\left[\varphi_{sam}(\omega )-\varphi_{ref}(\omega )\right]+1$$

(2) $$\alpha (\omega )=-\frac{d}{2}\ln\left\{\frac{{\left[n(\omega )+1\right]}^{2}}{4n(\omega )}\left|\frac{E_{sam}(\omega )}{E_{ref}(\omega )}\right|\right\}$$

where *c* is the speed of light in a vacuum, *ω* is the frequency of the THz wave, *d* is the thickness of the object being measured, $\left[\varphi_{sam}(\omega )-\varphi_{ref}(\omega )\right]$ signifies the phase difference between the sample signal and the reference signal, $E_{sam}(\omega )$ and $E_{ref}(\omega )$ are the frequency amplitudes obtained through FFT of the sampled signal and reference signal.

## 3 Results and analysis

### 3.1 THz absorption spectra of solid oridonin and ponicidin

**Figure 4:**
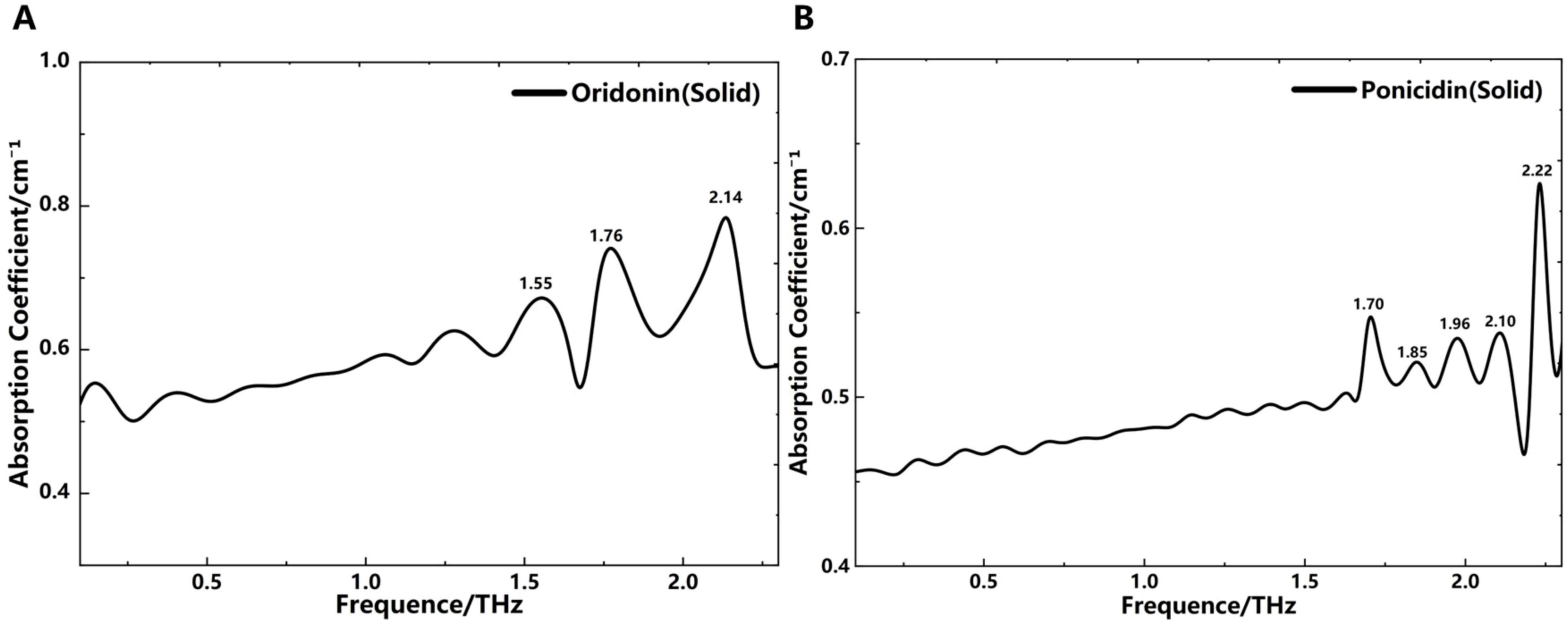
THz absorption spectra. **(A)** Oridonin (solid). **(B)** Ponicidin (solid).

Using Fast Fourier Transform, the THz absorption spectra of solid oridonin and ponicidin were obtained, as shown in **Figure 4A**. It can be observed that oridonin exhibits three distinct absorption peaks within the 0.1-2.3 THz range. The intensity variations at 1.55, 1.76, and 2.14 THz can be attributed to their different generation mechanisms. In contrast, ponicidin displays five absorption peaks at frequencies of 1.70, 1.85, 1.96, 2.10, and 2.22 THz, as illustrated in **Figure 4B**. These data reveal the unique spectral characteristics of oridonin and ponicidin.

### 3.2 THz absorption spectra of oridonin and ponicidin solutions

**Figure 5:**
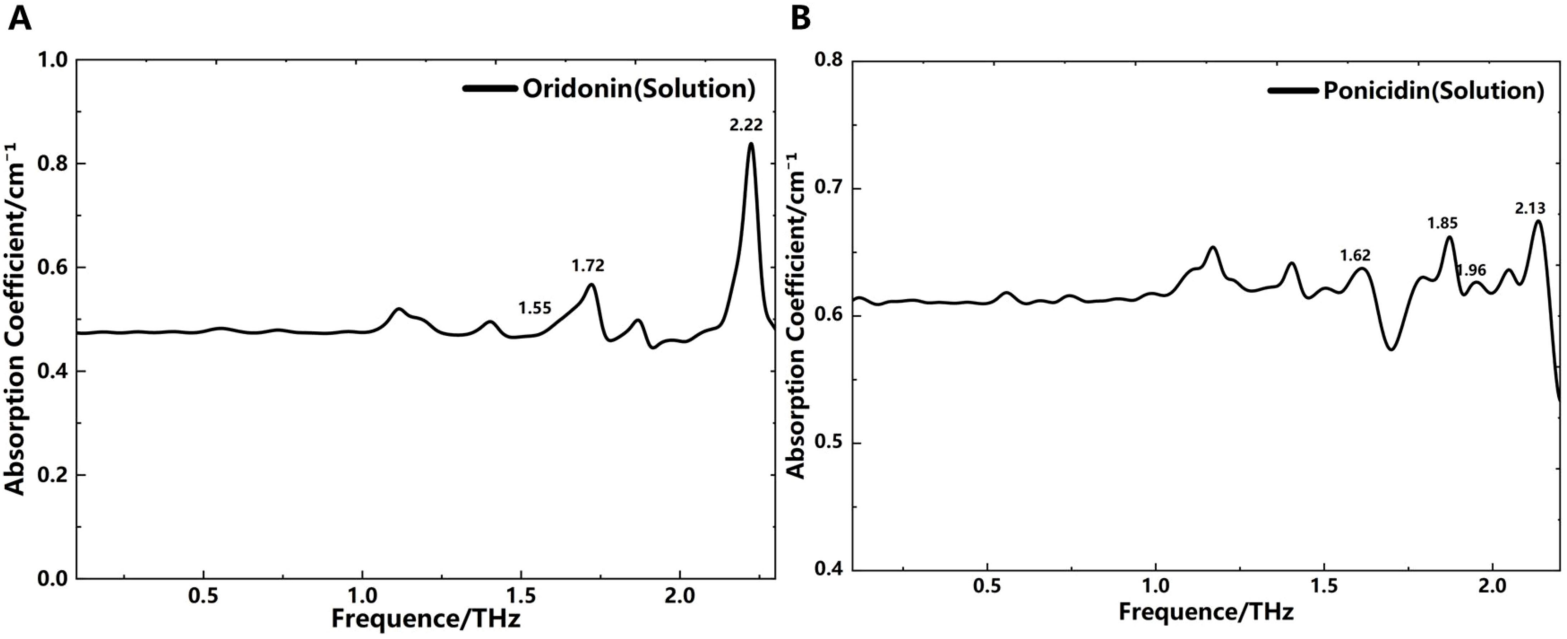
THz absorption spectra. **(A)** Oridonin (solution). **(B)** Ponicidin (solution).

Due to the poor water solubility of oridonin and ponicidin, we adopted ethanol as the solvent to measure the absorption spectra changes of these two substances in solution states. Meanwhile, to minimize the influence of ethanol liquid on THz wave absorption, we utilized microfluidic technology to control the thickness of the liquid layer to 50 μm. We then prepared oridonin-ethanol and ponicidin-ethanol solutions with maximum solubility and injected them sequentially into the microfluidic chip for measurement. The tests were conducted at a temperature of 25°C and a relative humidity below 2.6%. The resulting THz absorption spectra are shown in **Figure 5**. To obtain the liquid THz absorption spectra, we calculated the absorption coefficient of the samples using Formula 2, with ethanol solution replacing the empty chip as the reference signal for comparison. The THz absorption spectra of the solutions are presented in **Figure 5**. This data processing method effectively highlights the relative absorption peaks of the samples while minimizing the influence of the solvent, demonstrating its accuracy and effectiveness in capturing absorption peaks in liquid samples. However, due to ethanol’s strong absorption of THz waves, some absorption peaks differ from those in the solid state at certain THz frequencies. It can be observed that the absorption peaks of oridonin and ponicidin in solid and solution states are relatively consistent, but some peaks shift in frequency. Notably, the absorption peak of oridonin at 2.14 THz shifts slightly to the right in solution, while the absorption peak of ponicidin at 1.70 THz shifts slightly to the left.

These findings suggest that under the influence of ethanol, changes in intramolecular and intermolecular interactions cause slight variations in the absorption spectra between solid and solution states for oridonin and ponicidin. The reason for this phenomenon may be that ethanol disrupts or alters the hydrogen bonds, van der Waals forces, or other intermolecular interactions between the molecules of oridonin and ponicidin, thereby affecting the absorption spectra. In the solid state, the molecules are tightly packed and have specific orientations and interactions. When exposed to ethanol in the solution state, the molecules become more dispersed and interact in different ways. This may lead to shifts in absorption wavelengths or changes in absorption intensity. In addition, the intramolecular interactions between CH_3_/CH_3_, CH_3_/CH_2_ and CH_2_/CH_2_ can also lead to changes in the positions of some spectral bands (Romano et al., 2024). The shift in absorption peaks and the introduction of new absorption bands that are not present in the solid state reflect the significant impact of ethanol on the THz absorption spectra and the complex behavior of diterpenoids in solution environments. Meanwhile, this approach lays a foundation for studying the spectral characteristics of oridonin and ponicidin in solution environments.

### 3.3 Simulation of oridonin and ponicidin

**Figure 6:**
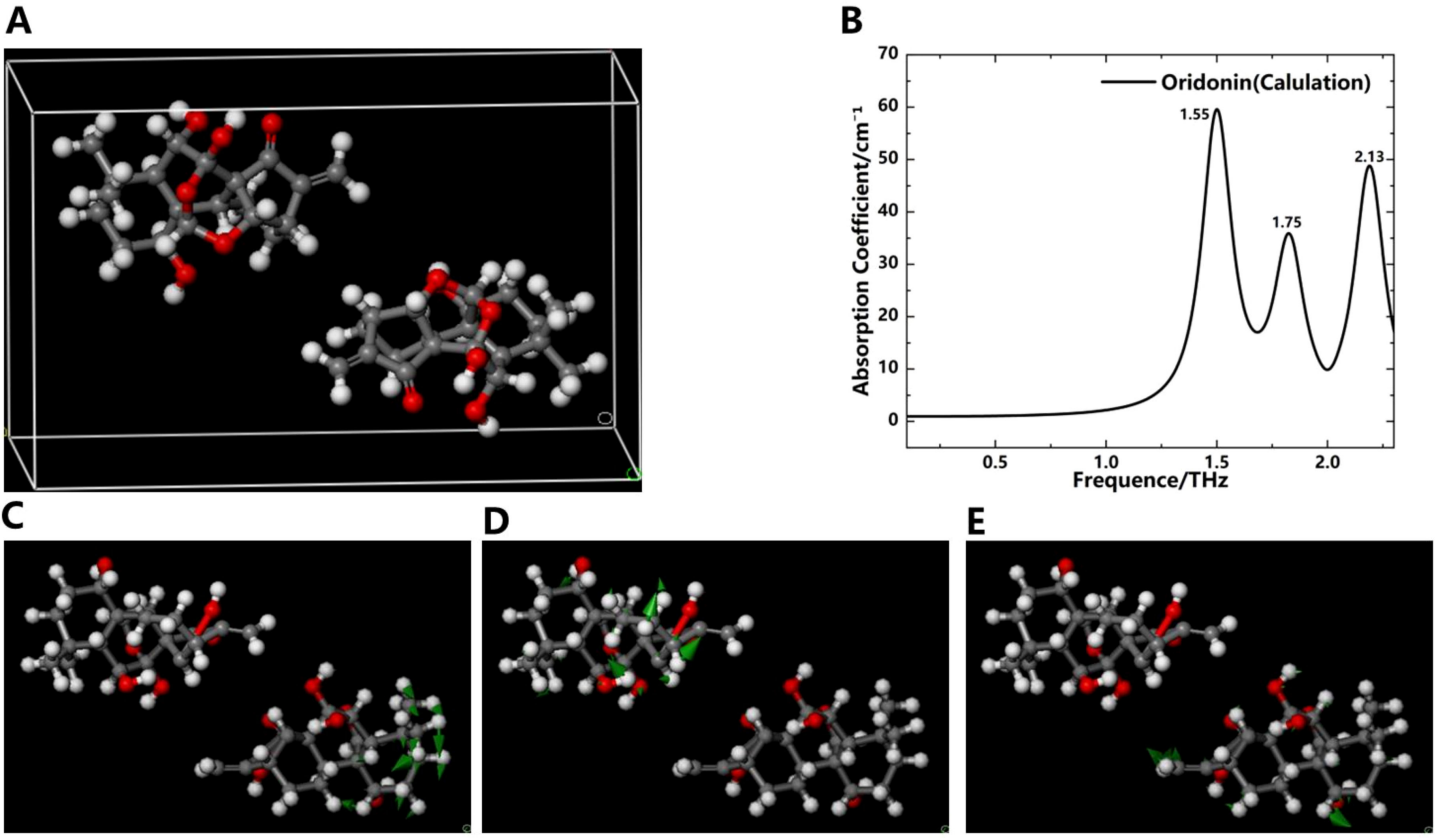
Simulation results of the THz absorption spectra and corresponding vibration modes of oridonin. **(A)** Crystal structure of oridonin. **(B)** Simulation spectrogram of oridonin. Vibration mode of oridonin at 1.55 THz **(C)**, 1.75 THz **(D)** and 2.13 THz **(E)**.

**Figure 7:**
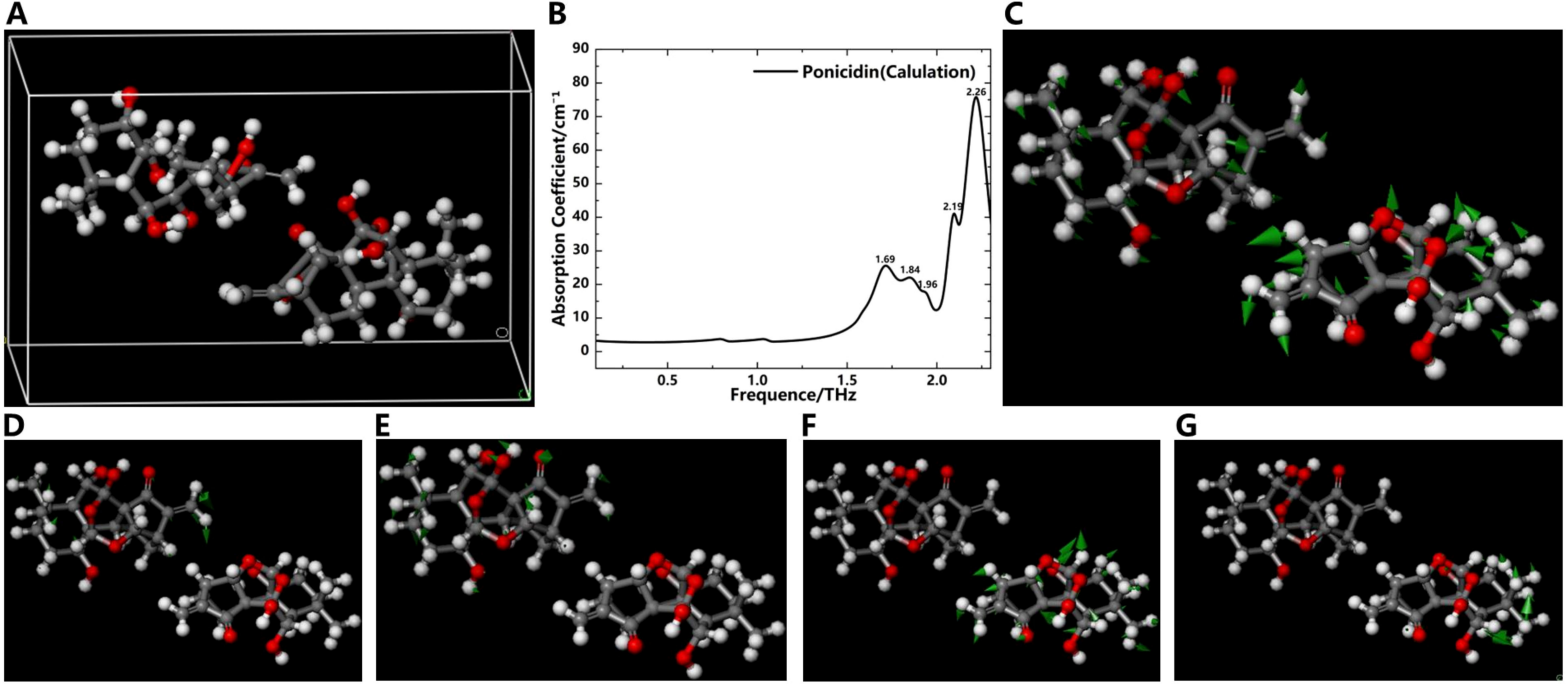
Simulation results of the THz absorption spectra and corresponding vibration modes of ponicidin. **(A)** Crystal structure of ponicidin. **(B)** Simulation spectrogram of ponicidin. Vibration mode of ponicidin at 1.69 THz **(C)**, 1.84 THz **(D)**, 1.96 THz **(E)**, 2.19 THz **(F)** and 2.26 THz **(G)**.

To further understand the origin of absorption peaks in the THz absorption spectra of oridonin and ponicidin, we employed DFT to investigate the factors influencing the absorption peaks of these compounds from the perspective of molecular forces. This approach allowed us to optimize the geometric structures of these molecules and calculate their vibrational modes (Bennett et al., 2019). The DFT-based calculation process using software such as Materials Studio mainly consists of two stages: geometric optimization followed by energy optimization using the Perdew-Burke-Ernzerhof (PBE)/Generalized Gradient Approximation (GGA) method. In **Figures 6** and **7**, we use the ball-and-stick model to describe the molecular structure. In this model, the dark gray balls represent carbon atoms, the white balls represent hydrogen atoms, and the red balls represent oxygen atoms. The single and double bonds between atoms are also graphically distinguished. In addition, in the molecular vibration diagram, green arrows are used to describe the direction of atomic vibrations.

**Figure 6A** depicts the crystal structure of oridonin. Its basic structure is a four-ring carbon skeleton, which contains a five-membered ring, a six-membered ring, a seven-membered ring, and a five-membered lactone ring. Four hydroxyl groups, one carbonyl group, and several alkyl functional groups are connected to different carbon atoms on the rings. Simulation results indicate that the absorption peaks of oridonin align closely with experimental data, appearing at frequencies of 1.55, 1.75, and 2.13 THz in **Figure 6B**. In addition, the absence of imaginary frequencies in the calculations indicates that the geometric optimization was highly successful, achieving a minimum energy structure during the modeling process. The vibration modes of oridonin are illustrated in **Figures 6C-E**. Through analysis, it was determined that the absorption peak at 1.55 THz primarily originates from the vibrations of the -CH_2_ and -CH_3_ groups, the peak at 1.75 THz is mainly due to the vibrations of the -OH group, and the peak at 2.13 THz stems from collective molecular vibrations. It’s worth noting that intermolecular interactions, especially hydrogen bonding, can significantly impact THz absorption peaks, potentially leading to slight discrepancies between simulated and experimental peaks. For instance, there are slight deviations between the simulated peak at 1.75 THz and the experimental peak at 1.76 THz, as well as between the simulated peak at 2.13 THz and the experimental peak at 2.14 THz.

**Figure 7A** shows the crystal structure of ponicidin. Similar to oridonin, its basic structure is also a four-ring carbon skeleton. The difference is that there is one more oxygen atom on the ring than in oridonin. At the same time, three hydroxyl groups, one carbonyl group, and several alkyl functional groups are connected to different carbon atoms on the rings. Simulation results demonstrate that the absorption peaks of ponicidin align reasonably well with experimental data, appearing at frequencies of 1.69, 1.84, 1.96, 2.19, and 2.26 THz, as illustrated in **Figure 7B**. The absence of any negative frequencies in the calculations underscores the accuracy of the simulated data. The vibration modes of ponicidin are presented in **Figures 7C-G**. According to the simulation results, the absorption peaks at 1.69, 1.96, and 2.19 THz are primarily attributed to collective molecular vibrations, while the peak at 1.84 THz originates from the vibrations of the -CH_2_, -CH_3_, and -OH groups. The peak at 2.19 THz again stems from collective molecular vibrations, and the peak at 2.26 THz is generated by the vibrations of various groups and intermolecular interactions. It’s worth noting that there are also slight discrepancies between the simulated and experimental absorption peaks, specifically between the simulated peak at 2.19 THz and the experimental peak at 2.10 THz, as well as between the simulated peak at 2.26 THz and the experimental peak at 2.22 THz. These discrepancies primarily arise from intermolecular interactions. Through a comparative analysis of experiments and simulations, we have verified the accuracy of the absorption peaks obtained in the experiments and analyzed the reasons for the appearance of each peak. This underscores the intricate influence of molecular vibrations on THz absorption peaks in oridonin.

In this study, several factors contributed to the discrepancies between experimental and simulated results. Firstly, the computational model is based on an idealized crystal structure, which may not accurately reflect the complexities encountered in the experimental environment. In practice, replicating an ideal crystal structure is often a challenging task. Secondly, the absorption peaks of THz radiation are significantly influenced by intermolecular interactions, particularly hydrogen bonding. Although these interactions were considered in the simulations, they may not have been fully or accurately represented, leading to slight differences in the positions of absorption peaks between simulated and experimental results. In summary, variations between experimental and simulated results arise from the idealization of the crystal structure and the complexity of intermolecular interactions. **Table 1** provides a comprehensive summary of the vibrational characteristics of oridonin and ponicidin in the THz frequency range, enabling a clearer understanding of the unique vibrational modes of these two substances in the THz absorption spectra.

**Table 1:** THz vibration modes of Oridonin and Ponicidin.

Sample	Experimental result (f/THz)	Theoretical simulation (f/THz)	Vibration mode attribution
Oridonin	1.55	1.55	-CH2, -CH3 in-plane bending vibration
	1.76	1.75	Hydrogen bond vibration -OH in-plane bending vibration
	2.14	2.13	Molecular collective vibration
Ponicidin	1.7	1.69	Molecular collective vibration
	1.85	1.84	-CH2, -CH3, -OH in-plane bending vibration
	1.96	1.96	Molecular collective vibration
	2.1	2.19	Molecular collective vibration
	2.22	2.26	Intermolecular interaction

### 3.4 Powder X-ray Diffraction

**Figure 8:**
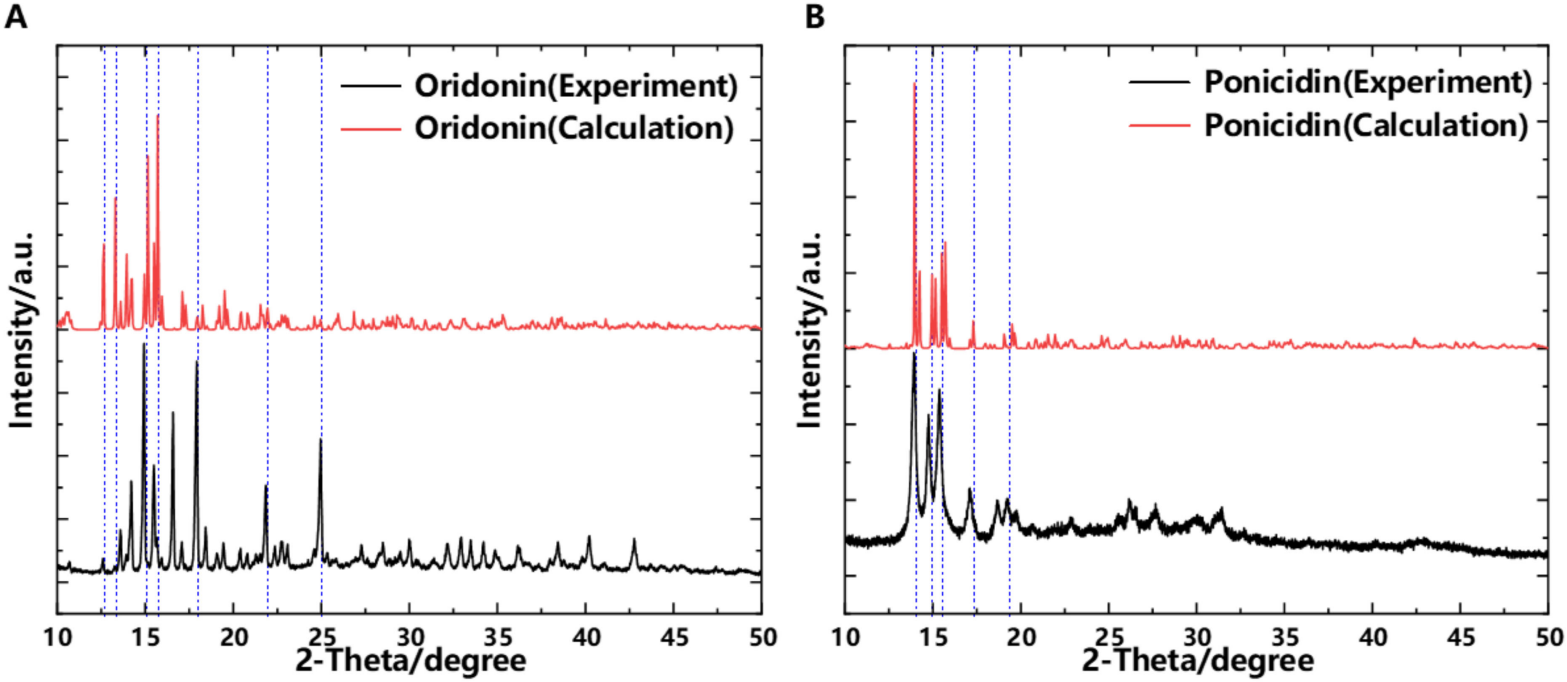
PXRD. **(A)** Oridonin. **(B)** Ponicidin.

To verify the accuracy of the collected data, PXRD (Powder X-ray Diffraction) experiments were conducted on the samples. PXRD experiments can provide detailed information about the crystal structure of the samples, helping us to accurately analyze the microstructure and lattice characteristics of the material. Through in-depth study of the PXRD patterns, important details such as crystal type, lattice parameters, and crystal orientation can be determined, which are significant for understanding the performance and nature of the material (Zhang et al., 2024). **Figure 8** presents the experimentally measured and simulated PXRD spectra of the samples. Through in-depth analysis and comparison of the PXRD experimental results with the simulation effects, we are pleased to find a high degree of consistency between the two. In the PXRD experiment, the obtained data clearly showed specific characteristic peaks, and the simulation results exhibited strong agreement with these experimental characteristic peaks in terms of position, intensity, and shape. This conclusion strongly demonstrates the high accuracy and reliability of our theoretical model and simulation method. It implies that our understanding of the structure and characteristics of related substances is correct, and it also verifies the accuracy of THz spectroscopy experiments and simulations. This provides a solid foundation for further research and application.

### 3.5 Raman spectroscopic characterization

Raman spectroscopy is a scattering spectroscopy based on the Raman scattering effect. By analyzing the scattering spectra with frequencies different from the incident light, it obtains information about molecular vibration and rotation, which is applied to the study of molecular structure. Raman spectroscopic analysis does not require sample preprocessing or preparation, thus avoiding some errors. It enables non-destructive qualitative and quantitative analysis, offering advantages such as simple operation, short measurement time, and high sensitivity during the analytical process (Jain et al., 2024). It typically operates in the near-infrared spectra, analyzing molecular vibration and rotation states. However, it is not as sensitive as THz spectroscopy in detecting the overall molecular vibration modes and intermolecular vibration modes. In some cases, it may also be affected by fluorescence interference or other background noises, resulting in a relatively low signal-to-noise ratio. To explore the feasibility of qualitative and quantitative analysis of oridonin and ponicidin using Raman spectroscopy, we attempted to analyze them using Raman spectroscopy after analyzing their absorption spectra with THz technology, and compared the analysis results of the two techniques.

**Figure 9:**
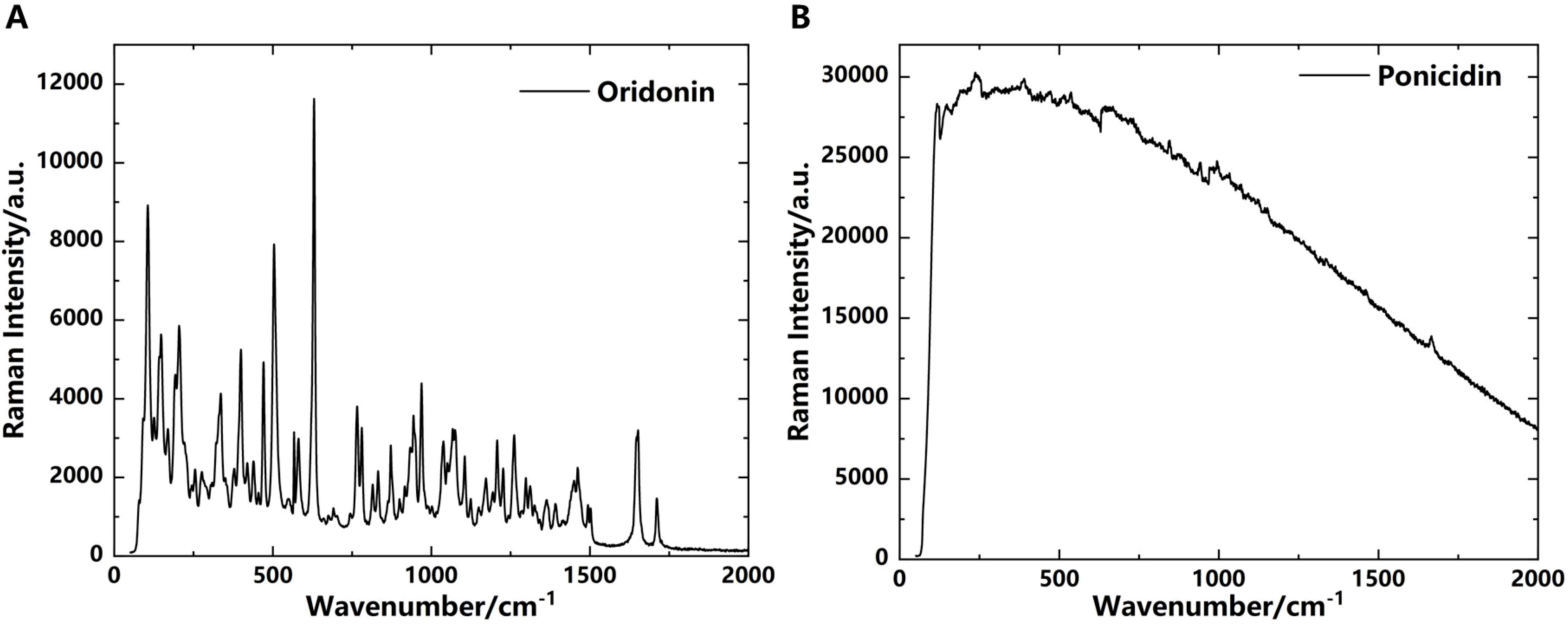
Raman spectroscopy. **(A)** Oridonin. **(B)** Ponicidin.

As shown in **Figure 9A**, the Raman spectroscopy of oridonin exhibits multiple distinct characteristic absorption peaks in the 0-2000 cm^-1^ waveband. The peak at 1711 cm^-1^ corresponds to the carbonyl C=O stretching vibration, the peak at 1652 cm^-1^ represents the C=C stretching vibration, the peaks between 1300-1500 cm^-1^ belong to the bending vibration of C-H in methyl and methylene groups, the peaks from 1200-1300 cm^-1^ are attributed to the ring C-O-C stretching vibration, the peaks in the range of 1000-1200 cm^-1^ correspond to the C-OH stretching vibration, the peaks from 780-1000 cm^-1^ represent the in-plane bending vibration of =C-H in unsaturated hydrocarbon groups, and the peaks between 400-780 cm^-1^ are attributed to the out-of-plane bending vibration of C-H in saturated hydrocarbon groups. However, the Raman spectroscopy of ponicidin does not show distinct characteristic absorption peaks in the 0-2000 cm^-1^ waveband in **Figure 9B**. After multiple measurements to eliminate experimental errors, it is possible that ponicidin, due to its internal structure and specific molecular vibration mode, produces a fluorescence effect when excited by light, resulting in a flat Raman spectroscopy in the 0-2000 cm^-1^ waveband, meaning there are no obvious absorption peaks. In contrast, both substances exhibit unique absorption peaks in their THz absorption spectra, which can be used for subsequent qualitative and quantitative analysis of oridonin and ponicidin in pharmaceuticals.

### 3.6 THz absorption spectra of Rabdosia rubescens capsules

**Figure 10:**
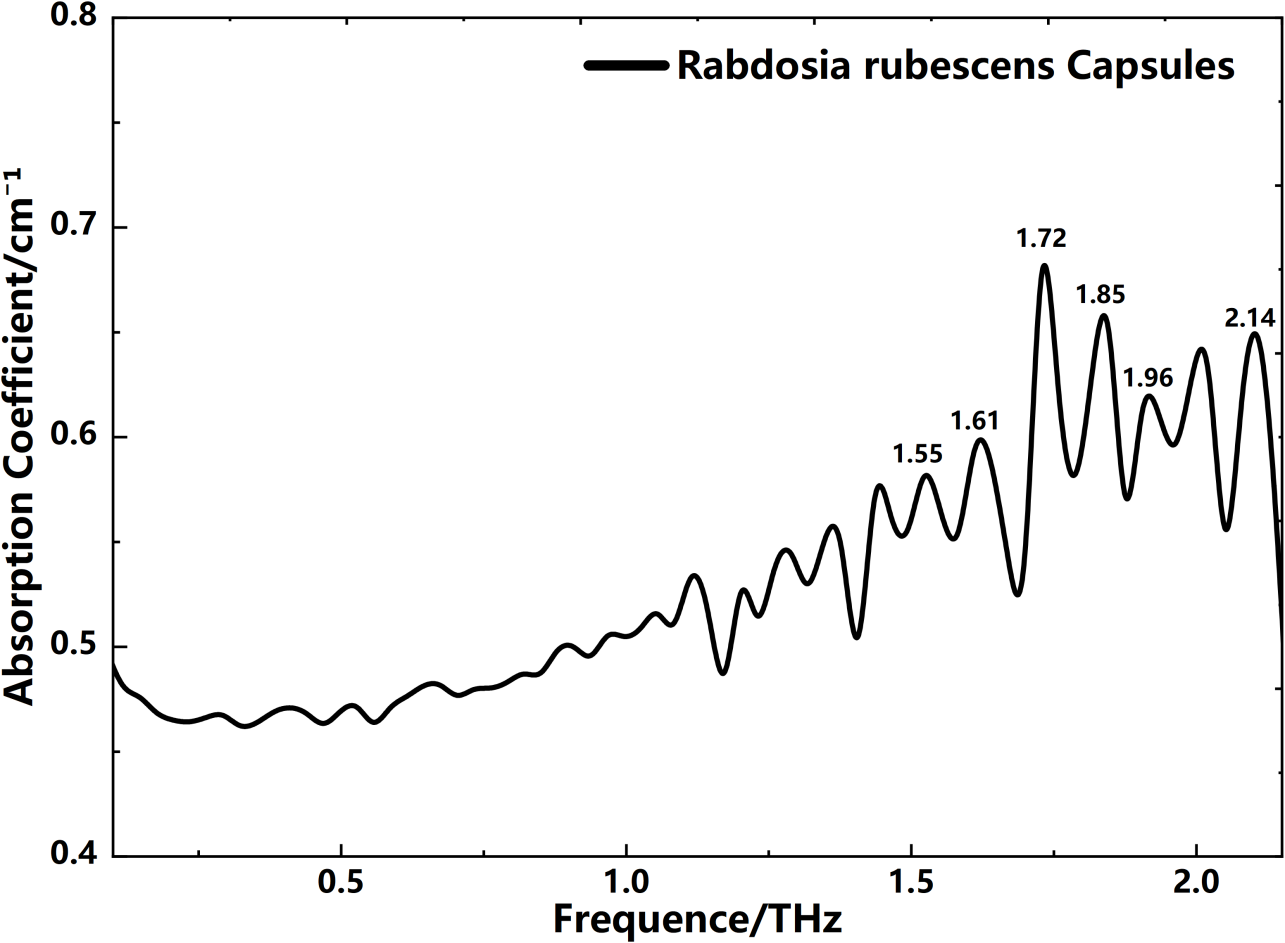
THz absorption spectra of Rabdosia rubescens Capsule.

In order to verify the accuracy of the experiment and explore the feasibility of THz methods in detecting the active ingredients of Rabdosia rubescens drugs, we conducted THz absorption spectra measurements on Rabdosia rubescens Capsule after obtaining the THz absorption spectra of oridonin and ponicidin. The THz absorption spectra obtained are shown in **Figure 10**. Since most of the oridonin-related medicines available on the market are direct extracts of oridonin, containing various chemical substances from the plant, the THz absorption spectra shows multiple absorption peaks. However, upon analysis, it was found that the absorption peaks at 1.55, 1.76, and 2.14 THz in oridonin can be clearly correlated in the THz absorption spectra of Rabdosia rubescens Capsule. Therefore, the THz absorption spectra can be used for the detection of oridonin in medicinal materials. Similarly, the absorption peaks of ponicidin at 1.70, 1.85, 1.96, and 2.14 THz can also be correlated in the THz absorption spectra of Rabdosia rubescens Capsule. Some different absorption peaks are due to the fact that other substances contained in Rabdosia rubescens Capsule can also absorb THz waves to varying degrees. Therefore, selecting appropriate absorption peaks is a key issue for subsequent quantitative analysis. Experimental results indicate that THz absorption spectra can be used for the detection of oridonin and ponicidin in Rabdosia rubescens Capsule, laying a foundation for subsequent quantitative analysis of the two substances based on peak values or peak areas.

## 4 Conclusion

In this study, the vibrational absorption spectra of oridonin and ponicidin was investigated using a THz-TDS system and DFT. Through discussions on the vibrational absorption characteristics of these two diterpenoids, distinct THz absorption peaks were identified. Simulation calculations were performed to obtain optimized stable structures and vibrational features, verifying the accuracy of the experimental data and showing good agreement with the experimental results. Additionally, microfluidic chip technology was utilized to measure the spectral characteristics of these two substances in solution, and their spectra in solid and ethanol solution states were compared. It was observed that the THz absorption peaks in solid and liquid states were relatively consistent, but some absorption peaks shifted due to the influence of hydrogen bonding in the ethanol solution. Furthermore, the THz absorption spectra of Rabdosia rubescens Capsule was measured, confirming the presence of oridonin and ponicidin in the capsules. Therefore, THz spectra provides a new approach for detecting the active substances oridonin and ponicidin in oridonin, offering a novel method for quality identification of the Chinese herbal medicine oridonin and laying a foundation for the application of THz in the biological research of Chinese herbal medicines.

## Data Availability

The raw data supporting the conclusions of this article will be made available by the authors, without undue reservation.
